# Impact of placental pathology on the risk of bronchopulmonary dysplasia in preterm infants: The role of gestational age and sex

**DOI:** 10.1007/s00431-025-06016-9

**Published:** 2025-02-26

**Authors:** C. Ramos-Navarro, R. Gregorio-Hernández, A. Pérez-Pérez, E. Rodríguez-Corrales, S. Vigil-Vázquez, M. Arriaga-Redondo, A. Merino-Hernández, M. Sánchez-Luna

**Affiliations:** Neonatology Department, Gregorio Marañón University Hospital, Madrid, Spain

**Keywords:** Bronchopulmonary dysplasia, Placental inflammation, Placental vascular malperfusion, Chorioamnionitis, Preterm birth

## Abstract

To analyze the impact of placental histological findings on the development of bronchopulmonary dysplasia (BPD) in preterm infants, this prospective, observational, single-center study included infants born before 32 weeks of gestation between 2012 and 2023. Perinatal variables were collected and correlated with mortality at hospital discharge and the diagnosis of grade 2–3 BPD at 36 weeks postmenstrual age (PMA). Placental histology was categorized into three groups: inflammatory pathology, vascular malperfusion, and no pathology. A total of 1128 preterm infants were enrolled, with placental histology results available for 899 cases. Inflammatory placental pathology was associated with a lower gestational age (GA) at birth (− 1.4 weeks, 95% *CI* − 1.74 to − 1.11). The increased mortality linked to placental inflammation was no longer significant after adjusting for GA. In preterm infants born at 27 weeks’ GA or later, the effect of vascular malperfusion on BPD showed sexual dimorphism. In males, placental malperfusion was associated with a 2.25-fold increased risk of developing BPD (95% *CI* 1.10 to 4.57), independent of GA and exposure to mechanical ventilation. No significant differences were observed in females born at 27 weeks or later.

*Conclusions*: The impact of placental histological abnormalities on BPD development is influenced by gestational age and sex. While placental inflammation increases mortality by triggering extremely preterm birth, it does not appear to increase respiratory morbidity compared to cases with normal placental histology at similar GAs. In males, however, placental malperfusion appears to affect lung development and contributes to BPD independently of GA and exposure to mechanical ventilation.

**Table:** 

**What is Known:** • *Bronchopulmonary dysplasia (BPD) is a common respiratory complication among preterm infants, strongly influenced by prenatal events.* • *The placenta plays a crucial role in fetal lung development, and its analysis provides objective insight into antenatal conditions.*	
**What is New:** • *Placental malperfusion affects lung development in a sex-specific manner, with male infants born at or after 27 weeks of gestation being more specifically affected and showing a higher susceptibility to BPD, independent of gestational age or mechanical ventilation.* • *These findings highlight the importance of considering sex differences in BPD pathophysiology and the role of placental pathology.*

**Supplementary Information:**

The online version contains supplementary material available at 10.1007/s00431-025-06016-9.

## Introduction

Bronchopulmonary dysplasia (BPD) is a prevalent respiratory complication among preterm newborns [1], with significant long-term morbidity implications [2]. This condition is characterized by an interruption in normal lung development, leading to structural and functional abnormalities. Its etiology is multifactorial and strongly influenced by prenatal events [3], with preterm birth representing the primary risk factor. Premature birth exposes the immature lung to postnatal stressors at an early stage of its development, exacerbating developmental abnormalities that often originate in the prenatal period.

The placenta plays a fundamental role in fetal development, performing essential functions such as gas exchange, nutrient transport, waste elimination, and regulation of the maternal–fetal immune response [4]. In addition, the placenta is actively involved in maternal–fetal communication through the release of exosomes, which modulate fetal gene expression and might influence neonatal susceptibility to various diseases throughout life [5–7]. In particular, significant structural and functional similarities have been identified between the placenta and the developing lung, suggesting that prenatal events or insults may affect both organs in similar ways [3].

Placental pathology can be categorized into two main groups: inflammatory pathology, which includes acute or chronic inflammatory changes and vascular pathology, which can be of either maternal or fetal origin [8, 9].

Placental vascular malperfusion is one of the primary precipitants of preterm birth and has also been identified as a prenatal factor associated with the development of BPD, independent of the degree of prematurity [10] [11].

Histological chorioamnionitis has been extensively studied due to its frequent association with prematurity; however, its role in respiratory morbidity remains unclear, with conflicting results. Some studies have even suggested a protective effect against the incidence of respiratory distress syndrome in preterm infants [12] [13]. The relationship between chorioamnionitis and BPD is also controversial, with studies reporting divergent findings. This variability is partly due to the frequent association of chorioamnionitis with extreme prematurity, which is the primary risk factor for the development of BPD [14] [15].

Structural or functional abnormalities of the placenta not only affect fetal development but are also frequently associated with the onset of preterm labor. Examination of the placenta provides critical insight into the mechanisms underlying preterm birth and the intrauterine environment that may have influenced the preterm newborn. Moreover, understanding the relationship between these placental abnormalities and postnatal outcomes can provide valuable information to optimize and personalize the clinical management of preterm infants [16].

The aim of this study is to evaluate the impact of placental histological abnormalities on mortality and the development of BPD in our population of preterm infants.

## Materials and methods

### Study design

This was a single-center, analytical, observational study conducted in the Gregorio Marañón Neonatal Intensive Care Unit (NICU). The study period extended from January 2012 to December 2023.

### Study population

The study included all preterm infants born before 32 weeks of gestation who were admitted to the NICU within the first 6 h of life during the specified period. Exclusion criteria were newborns with severe congenital anomalies and those who died in the delivery room.

### Data collection

The following perinatal variables were recorded: *gestational age at birth* (GA) (determined in weeks and days from the last menstrual period, or if unavailable, estimated via first trimester ultrasound), *birth weight* (in grams), *sex*, and *administration of antenatal corticosteroids*. A complete course of corticosteroids was defined as two doses of betamethasone (totaling 24 mg of Celestone™) administered to the mother between 24 h and 7 days before delivery. Additional perinatal respiratory variables were documented, including the *need for intubation* within the first 10 min of life, *administration of surfactant*, and *time of mechanical ventilation* during the first 3 days of life and *total duration of MV during hospitalization*.

### Histological examination of the placenta

The primary focus of this study was the histological examination of the placenta. Placental samples were collected at the time of delivery and subsequently processed for histological analysis. Sections were prepared using paraffin embedding and stained with hematoxylin and eosin. All histological analyses were conducted by an experienced pathologist located at the study center, specializing in placental evaluation. The pathologist performed detailed histological assessments, and the corresponding descriptive reports were included in the institutional database. These histological descriptions were later categorized retrospectively according to the 2015 Amsterdam criteria [8] for placentas analyzed prior to 2016. When additional clarification was required, maternal medical records and original pathology reports were reviewed to ensure accurate classification.

From 2016 onward, the classification of placental lesions was performed prospectively according to the 2015 Amsterdam criteria, with the results systematically recorded in the institutional database. Due to the relatively small number of placentas (45 cases) meeting the criteria for fetal vascular lesions, maternal and fetal vascular lesions were combined into a broader category of vascular malperfusion to ensure robust analysis and facilitate interpretation of the data. This regrouping resulted in the creation of three distinct categories:

- Placental histology without pathological findings,
- Placental histology with signs of vascular malperfusion, and
- Placental histology with signs of inflammation.

In cases where the placenta showed normal histology or was not examined, the obstetric history from the maternal medical records was reviewed to identify potential triggers for preterm birth under these conditions.

### Primary outcome

The primary outcome of this study is survival to hospital discharge without a diagnosis of grade 2 or 3 BPD. The classification of BPD is based on the National Institutes of Health (NIH) consensus definition [17], considering only grades 2 and 3, which are characterized by the need for respiratory support (either positive pressure or supplemental oxygen) for at least 28 days and ongoing support at 36 weeks postmenstrual age (PMA). For cases requiring less than 30% oxygen at 36 weeks PMA, a reduction test is performed to confirm whether supplemental oxygen is still necessary [18]. This outcome is referred to as “survival free of BPD” (SFBPD).

### Data correlation

An initial descriptive analysis was conducted on the entire population of premature newborns under 32 weeks of gestation who were admitted to the unit during the study period.

Subjects for whom histological analysis of the placenta was unavailable were excluded from the subsequent correlation study between placental histological findings and the development of mortality and grade 2–3 BPD.

The data were analyzed using IBM SPSS for Mac, version 26 (Chicago, Illinois). Descriptive statistics were presented as measures of central tendency and dispersion for quantitative variables (median and interquartile range (*IQR*)) and as percentage distributions for qualitative variables. For bivariate analyses involving multiple comparisons, the chi-square test (*χ*^2^) with Bonferroni correction was applied to qualitative variables. Since the quantitative variables did not follow a normal distribution (as confirmed by the Kolmogorov–Smirnov test), non-parametric tests were employed for group comparisons. Specifically, the Kruskal–Wallis test with Bonferroni correction was used for multiple comparisons of distributions. Statistical significance was defined as *p* < 0.05.

The multivariate analysis included both linear regression and binary logistic regression. First, an unadjusted analysis was conducted to evaluate the direct correlation between placental histology and the primary outcome, SFBPD. This was followed by an adjusted analysis, in which variables were selected based on their theoretical relevance and statistical significance identified in the bivariate analysis. We aimed to avoid including variables that might act as intermediates in the causal pathway between placental abnormalities and neonatal outcomes, as this could obscure the direct relationships of interest. Therefore, our initial model was adjusted for gestational age [19], prenatal corticosteroid administration [20], and sex [21, 22]. We also assessed multicollinearity among these variables by calculating the variance inflation factor (*VIF*) for each variable. The *VIF* values were below 1.5, indicating no significant multicollinearity. Linear regression was used to assess the association between GA and placental histology. Additionally, interactions between the independent variable and covariates were tested, and stratification was applied when significant interactions were identified. The Hosmer–Lemeshow test was used to assess the model’s goodness-of-fit.

The GA cutoff was determined based on the gestational age with the highest overall predictive capacity for SFBPD and mortality, as assessed through area under the curve (*AUC*) analysis.

## Results

During the study period, a total of 1128 neonates born at less than 32 weeks of gestation were admitted to the unit. The median GA was 28.5 weeks (*IQR* 26.4–30.6), and the median birth weight was 1080 g (*IQR* 800–1380). Overall, 89.5% of neonates received at least one dose of antenatal corticosteroids, with 62.2% completing a full treatment course. A descriptive analysis according to placental histological findings is presented in Table 1.

**Table 1:** Perinatal characteristics of the total sample (n1128) according to the placental histology group

	No pathology (n235)	Inflammation (n267)	Malperfusion (n397)	Not analyzed (n229)	*p*
Gestational age (weeks)	28.6 (26.8–30.3)	27.0 (25.4–29.0)	29.3 (27.0–30.7)	29.8 (27.7–31.19	< 0.01*
• < 27 wGA (331, 29.3%)	63 (26.8%)	129 (48.3%)	96 (24.2%)	43 (18.8%)	< 0.01^†^
Birth weight (grams)	1100 (830–1430)	960 (740–1230)	1040 (780–1340)	1270 (1000–1527)	< 0.01^‡^
Female sex	89 (37.9%)	121 (45.3%)	189 (47.6%)	101 (44.1%)	0.113
Antenatal corticosteroids (ANS)	207 (88.1%)	246 (92.1%)	366 (92.2%)	191 (83.4%))	< 0.01^§^
• ANS complete course	136 (57.9%)	171 (64%)	260 (65.5%)	135 (59%)	0.153
Intubation in delivery room	76 (32.3%)	110 (42.1%)	107 (27%)	51 (22.3%)	< 0.01**
Surfactant	149 (63.4%)	151 (56.6%)	220 (55.4%)	113 (49.3%)	0.024^††^
MV in the first 3 dol	99 (42.1%)	115 (43.1%)	157 (39.5%)	85 (37.1%)	0.527
Duration of MV in the first 3 dol	0 (0–48)	0 (0–48)	0 (0–24)	0 (0–34.5)	0.033^‡‡^
Total duration of MV during hospitalization (hs)	8 (0–183.5)	24 (0–192)	1 (0–120)	0 (0–105)	< 0.01^§§^
BPD 2–3; n203 (20.9%)	44 (22%)	53 (25%)	76 (22%)	30 (14%)	0.032^***^
Mortality; n56 (13.8%)	35 (14.9%)	55 (20.6%)	52 (13.1%)	14 (6.1%)	< 0.01^†††^

*Significant differences between all comparations, ** significant differences between inflammation and malperfusion, and inflammation and not analyzed, *** significant differences between not analyzed and inflammation

^**†**^Significant differences only between inflammation an all the other groups

^‡^Significant differences between all comparations except normal and not analyzed: 0.056

^§^Significant differences between inflammation and not analyzed and between malperfusion and not analyzed

^††^Significant differences between normal and not analyzed

^**‡‡**^Significant differences between malperfusion and normal and malperfusion and inflammation

^§§^Significant differences between normal and not analyzed, inflammation and not analyzed, and between inflammation and malperfusion

^†††^Significant differences between not analyzed and the other groups

The Supplementary Information 1 provides information on the potential mechanisms that contributed to premature birth in cases where the placenta was either not examined or no pathological alterations were identified.

For the correlation analysis between placental histology and outcome variables, 229 subjects without available placental histological results were excluded (Supplementary Information 2).

In the unadjusted analysis, placental inflammatory pathology was associated with reduced SFBPD (*OR* 0.66; 95% *CI* 0.49–0.87), primarily due to an increase in mortality (*OR* 1.80; 95% *CI* 1.25–2.59). However, this association was no longer significant after adjusting for GA (*OR* 0.80; 95% *CI* 0.51–1.23).

In contrast, placental malperfusion did not show a significant effect on SFBPD in the unadjusted analysis (*OR* 1.25; 95% *CI* 0.94–1.65). However, after adjusting for GA, an association emerged indicating a reduction in SFBPD (*OR* 0.67; 95% *CI* 0.47–0.95). This effect was primarily driven by an increased incidence of grade 2–3 BPD (*OR* 1.53; 95% *CI* 1.03–2.28).

**Fig. 1:**
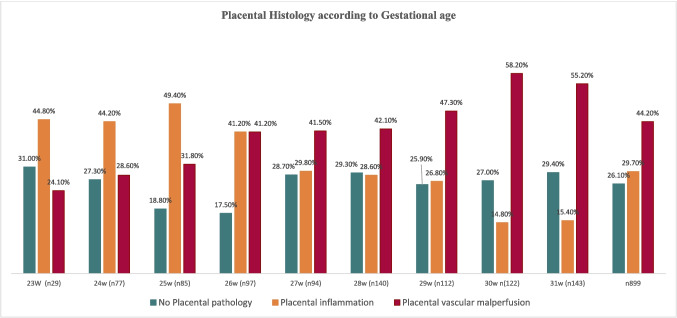
Placental histology findings by gestational age (weeks)

Significant differences in perinatal characteristics were observed among groups with different placental histologies, particularly GA. Cases with inflammatory histology had a median GA at birth that was 1.42 weeks lower (95% *CI* − 1.74 to − 1.10) compared to the other two groups combined. Moreover, the proportion of infants born before 27 weeks was twice as high in the inflammatory histology group compared to those with vascular malperfusion (*OR* 2.92; 95% *CI* 2.10–4.08) (Table 1). Figure 1 illustrates placental histological findings according to GA. In this study, each additional week of GA nearly doubled the likelihood of SFBPD (*OR* 1.95; 95% *CI* 1.79–2.15), regardless of placental histology. Additionally, being born before 27 weeks was associated with a 13.9-fold increase in mortality risk (95% *CI* 9.1–21.4).

Given the significant differences in GA between histological groups and its strong association with SFBPD (Supplementary Information 3), a stratified analysis by GA groups was conducted. Based on the observation that 27 weeks showed the highest predictive capacity, with *AUC* values of 0.75 (95% *CI* 0.71–0.79) for SFBPD and 0.84 (95% *CI* 0.77–0.90) for mortality (Supplementary Information 4), a cutoff at 27 weeks of GA was established for this analysis (< 27 and ≥ 27 weeks).

When analyzing the impact of placental histology on SFBPD in neonates over 27 weeks of GA, an interaction between sex and placental histology was observed, specifically in cases of vascular malperfusion. This indicates that the effect of placental malperfusion on SFBPD differs between males and females. Therefore, an additional stratification by sex was performed. The results of this analysis are presented in Tables 2 and 3.

**Table 2:** Outcomes by placental histology in infants born at less than 27 weeks of gestational age, stratified by sex. Data are expressed as *n* (%) or *aOR* (95% *CI*)

		Female (122)					Male (166)					
		No pathology (27; 22.1%) GA (25.0; 24.0–26.2)	Inflammation (61; 50%) GA (25.4; 24.7–26.0)	Malperfusion (34; 27.9%) GA (26.1; 24.6–26.4)	*p*	aOR (GA, ANS)		No pathology (36; 21.7%) GA (25.0; 24.4–26.1)	Inflammation (68; 41%) GA (25.3; 24.0–26.1)	Malperfusion (62; 37.3%) GA (25.4; 24.6–26.3)	*p*	aOR (GA, ANS)
< 27 weeks GA	**SFBPD** **(38; 31.1%)**	8 (29.6%)	23 (37.7%)	7 (20.6%)	0.221	0.36 (0.10–1.32)*	**SFBPD (47; 28.3%)**	8 (22.2%)	20 (29.4%)	19 (30.6%)	0.649	1.25 (0.43–3.66)*
						**0.32** (**0.11–0.88)** *p* = **0.028**^†^						0.94 (0.40–2.23)^**†**^
						1.25 (0.43–3.62)^‡^						1.38 (0.47–4.05)^‡^
	**MORTALITY** **(42; 34.4%)**	11 (40.7%)	19 (31.1%)	12(35.3%)	0.677	1.28 (0.39–4.17)*	**MORTALITY (70; 42,2%)**	15 (41.7%)	30 (44.1%)	25 (40.3%)	0.907	1.31 (0.57–3.00)*
						1.52 (0.58–4.01)^†^						1.01 (0.45–2.26)^**†**^
						0.89 (0.31–2.56)^‡^						1.19 (0.47–3.04)^‡^
	**BPD 2–3** **(42; 52.5%)**	8 (50%)	19 (45.2%)	15 (68.2%)	0.212	2.71 (0.68–10.98)*	**BPD 2–3** **(49; 51%)**	13 (61.9%)	18 (47.4%)	18 (48.6%)	0.527	0.70 (0.22–2.25)*
						**3.18** **(1.02**–**9.91**) *p* = **0.046**^†^						1.08 (0.41–2.86)^†^
						0.83 (0.25–2.73)^‡^						0.64 (0.20–2.06)^‡^
	**Malperfusion**	**No** **(88; 72.1%)**		**Yes** **(34; 27.9%)**	***p***	**aOR (GA, ANS)**	**Malperfusion**	**NO** **(104; 62.7%)**		**YES** **(62; 37.3%)**	***p***	**aOR (GA, ANS)**
	**SFBPD** **(38; 31.1%)**	31 (35.2%)		7 (20.6%)	0.13	**0.32** **(0.12–0.89)** (*p* = **0.028**)	**SFBPD** **(47; 28.3%)**	28 (26.9%)		19 (30.6%)	0.72	1.04 (0.47–2.27)
	**MORTALITY (42; 34.4%)**	30 (34.1%)		12 (35.3%)	1.0	1.45 (0.57–3.64)	**MORTALITY (n70; 42.2%)**	45 (43.3%)		25 (40.3%)	0.75	1.11 (0.53–2.25)
	**BPD 2–3** **(42; 52.5%)**	27 (46.6%)		15 (68.2%)	0.13	**3.05** (**1.02–9.08)** *p* (**0.045**)	**BPD 2–3** **(n49; 51.0%)**	31 (52.5%)		18 (48.6%)	0.83	0.93 (0.38–4.6)

No significant differences in GA (median; *IQR*) were observed between placental histology groups (females: *p* = 0.052; males: *p* = 0.423).* GA* gestational age, *ANS* antenatal corticosteroids (complete course), *aOR* adjusted odds ratio, *CI* confidence interval

*Malperfusion vs. no pathology

^†^Malperfusion vs. inflammation

^‡^Inflammation vs. no pathology

**Table 3:** Outcomes by placental histology in infants born at ≥ 27 weeks of gestational age, stratified by sex. Data are expressed as *n* (%) or *aOR* (95% *CI*)

	Female (277)						Male (334)					
		No pathology (62; 22.4%) GA (30.0; 28.2–31.4)	Inflammation (60; 21.7%) GA (28.6; 27.7–29.5)	Malperfusión (155; 56%) GA (30.0; 28.6–31.0)	*p*	aOR (GA, ANS)		No pathology (110; 32.9%) GA (29.4; 28.1–30.7)	Inflammation (78; 23.4%) GA (29.3; 28.5–30.3)	Malperfusion (146; 43.7%) GA (29.7;28.5–31.0)	*p*	aOR (GA, ANS)
≥ 27 weeks GA	**SFBPD 2–3 (236; 82.5%)**	50 (80.6%)	50 (83.3%)	136 (87.7%)	0.34	1.73(0.74–4.05)*	**SFBPD 2–3 (263;78.7%)**	90 (81.8%)	66 (84.6%)	107 (73.3%)	0.09	0.51 (0.26–0.97)*p = **0.042**
						0.82(0.34–2.0)^†^						**0.38 (0.18–0.82)**^†^ *p* = **0.014**
						1.88 (0.68–5.18)^‡^						1.30 (0.57–2.94)^‡^
	**MORTALITY** **(11; 4%)**	4 (6.5%)	2 (3.3%)	5 (3.2%)	0.52	0.49 (0.12–1.93)*	**MORTALITY** **(19; 5.7%)**	5 (4.5%)	4 (5.1%)	10 (6.8%)	0.71	1.79(0.58–5.52)*
						1.34 (0.25–7.56)^†^						1.45(0.43–4.89)^†^
						0.41 (0.07–2.48)^‡^						1.26 (0.32–4.95)^‡^
	**BPD 2–3** **(30; 11.3%)**	8 (13.8%)	8 (13.8%)	14 (9.3%)	0.53	0.64 (0.23–1.72)*^*^	**BPD 2–3** **(52; 16.5%)**	15 (14.3%)	8 (10.8%)	29 (21.3%)	0.11	2.0 (0.96–4.2)*
						1.12 (0.44–3.12)^†^						**3.2 (1.31–7.83)**^†^ *p* =** 0.011**
						0.57 (0.18–1.84)^‡^						0.62 (0.24–1.62)^‡^
	**Malperfusion**	**NO** **(122; 44%)**		**YES** **(155; 56%)**	***p***	***aOR* (GA, ANS)**	**Malperfusion**	**NO** **(188; 56.3%)**		**YES** **(146; 43.7%)**	***p***	***aOR* (GA, ANS)**
	**SFBPD** **(266; 85.2%)**	100 (82%)		136 (87.7%)	0.23	1.18 (0.58–2.40)	**SFBPD** **(263; 78.7%)**	153 (83%)		107 (73.3%)	0.03	**0.45** **(0.26–0.80**) *p* **<** **0.01**
	**EXITUS** **(11; 4%)**	6 (4.9%)		5 (3.2%)	0.54	0.77 (0.22–2.65)	**MORTALITY** **(n19; 5.7%)**	9 (4.8%)		10 (6.8%)	0.48	1.65 (0.64–4.22)
	**BPD 2–3** **(30; 11.3%)**	16 (13.8%)		14 (9.3%)	0.33	0.89 (0.39–2.01)	**BPD 2–3** **(n52; 16.5%)**	23 (12.8%)		29 (21.3%)	0.04	**2.4 (1.2–4.6)** *p* <** 0.01**

Significant differences in *GA* (median; *IQR*) were observed among females (*p* < 0.01 between the inflammation group and the other two groups), while no significant differences were found among males.* GA* gestational age, *ANS*, antenatal corticosteroids (complete course), *aOR*, adjusted odds ratio, *CI*, confidence interval

*Malperfusion vs. no pathology

^†^Malperfusion vs. inflammation

^‡^Inflammation vs. no pathology

In neonates born at less than 27 weeks of gestation, the overall mortality rate was 38.9%, with 42.2% in males and 34.4% in females. Among survivors, the incidence of BPD 2–3 was 51.7%, with 52.5% in females and 51% in males. The incidence of SFBPD was 29.5%, with 31.1% in females and 28.3% in males.

No significant differences in mortality were found to be associated with placental abnormalities in either sex.

In females, placental vascular malperfusion was associated with an increased incidence of BPD 2–3, which led to a significant reduction in the incidence of SFBPD but only when compared to inflammation and after adjustment for GA. In the crude analysis, the differences did not reach significance (see Table 2).

In males, no significant associations were found between placental histology and any of the variables analyzed.

In neonates born at 27 weeks of gestation or later, the overall mortality rate was 4.9%, with 4% in females and 5.7% in males. The incidence of BPD 2–3 was 14.1%, with 11.3% in females and 16.6% in males, while the incidence of SFBPD was 81.7%, with 82.5% in females and 78.7% in males.

No significant differences in mortality were found to be associated with placental abnormalities in either sex.

In females, no significant differences were observed in any variables associated with placental abnormalities.

In males, placental malperfusion was associated with a higher incidence of BPD 2–3 and a lower likelihood of SFBPD compared to both inflammation and non-pathological histologies when grouped together. In the adjusted analysis, this association remained significant when compared independently to both no pathology and inflammation (see Table 3). The association between malperfusion and SFBPD remained significant even without adjusting for GA (OR 0.57; 95% *CI* 0.33–0.97; *p* = 0.037). Furthermore, this effect was independent of the duration of mechanical ventilation (*OR* 0.487; 95% *CI* 0.25–0.95; *p* = 0.03).

## Discussion

In our study population, we found that placental malperfusion was associated with an increased incidence of BPD 2–3 in male infants with a gestational age (GA) greater than 27 weeks.

### Association between placental malperfusion and BPD

The link between placental malperfusion and the development of BPD is well-documented, though the underlying mechanisms remain unclear [10, 11]. The placenta and lungs share similarities in morphology and function during fetal development, suggesting that alterations in intrauterine environment may impact both organs similarly [3].

Recent studies have shown a correlation between placental malperfusion and decreased levels of angiogenic factors in the umbilical cord of preterm infants [23]. These biomarkers have been identified as predictors of pulmonary hypertension associated with BPD, suggesting that impaired placental angiogenesis plays a key role in pulmonary vascular pathology [24]. This disruption in fetal angiogenesis not only affects pulmonary vascular development but also impairs alveolarization and lung growth [25].

Animal studies have demonstrated that inhibiting angiogenic factors disrupts both fetal vascular development and pulmonary alveolarization [26, 27], highlighting the critical link between vascular and alveolar development in the pathophysiology of BPD [28].

Altered angiogenic signaling plays a key role in the association between placental malperfusion and BPD, underscoring the importance of optimal angiogenesis for lung development [29].

Furthermore, changes in maternal blood microRNA profiles have been identified as potential biomarkers for placental dysfunction [30]. MicroRNAs are small RNA molecules produced in various tissues, including the placenta, that regulate gene expression. They play a crucial role in growth, differentiation, and vascular development. Specifically, the microRNA profile associated with intrauterine growth restriction is involved in processes that impact both angiogenesis and placental function [31], linking placental malperfusion with BPD development.

### Sexual dimorphism

Recent evidence suggests that maternal microRNA patterns may vary in response to placental insufficiency, depending on fetal sex [32] [33]. This phenomenon provides a potential explanation for the differing effects of placental malperfusion between females and males observed in our study.

Additionally, differences in microRNA expression in lung tissue between males and females during fetal development have been described, indicating that microRNAs may regulate sex-specific differences in lung development and contribute to varying susceptibility to postnatal pulmonary disease [34]. An animal model has shown that microRNAs’ expression in the lungs in response to hyperoxia differs by sex, with females exhibiting a protective effect on angiogenesis and alveolarization [35].

In this study, it was found that in female infants, placental alterations were not associated with increased respiratory morbidity at 36 weeks PMA when delivery occurred after 27 weeks GA. However, in female infants born before 27 weeks GA, a higher incidence of BPD was observed in cases of vascular malperfusion compared to placental inflammation, which reached statistical significance when adjusted for GA. Due to the small sample size and the complexity of interpreting the relationship between placental inflammation and GA, drawing definitive conclusions is challenging. One possible explanation could be that the impact of placental alterations varies according to the timing of gestation, as has been previously described for the effects of exposure to adverse events during pregnancy [36], with this impact potentially differing by sex. Placental malperfusion was associated with a higher incidence of BPD in male infants only when born after 27 weeks. It is known that, at the same gestational age, fetal lung development is structurally more mature in females than in males [37] and that females also exhibit a more developed antioxidant response at birth [38], which could explain their greater capacity for adaptation to prenatal placental alterations.

The fact that the effect of placental malperfusion on the development of BPD in males is independent of GA, and mechanical ventilation exposure (MV) suggests that these alterations are present at birth and impact lung independently of postnatal factors, such as MV, that are strongly associated with BPD [39].

The greater susceptibility of males to morbidity associated with prematurity, including the development of BPD, is well documented, indicating that fetal sex plays a crucial role in its pathophysiology [40, 41]. Placental sex differences exist from early prenatal development [42] which could contribute to the discrepancies in postnatal morbidity. Enhancing our understanding of the mechanisms behind these differences is essential for developing treatments targeting the specific molecular pathophysiological bases.

### Placental inflammation and gestational age

The relationship between placental inflammation and the incidence of BPD is complex, primarily due to the strong association between placental infection/inflammation and extremely preterm birth. A recent study shows that histological chorioamnionitis, a form of placental inflammation, increases the risk of BPD; however, this effect is largely mediated by the degree of prematurity [43]. In other words, chorioamnionitis increases the risk of BPD mainly by triggering extremely preterm birth rather than through inflammation itself. Moreover, since placental inflammation is a known cause of preterm labor, its inclusion as a confounding factor in analytical models is debatable [44].

In this study, placental inflammation was associated with a decrease SFBPD, but this effect was no longer significant after adjusting for gestational age. In subgroup analysis, placental inflammation did not increase the incidence of BPD or mortality in infants born before or after 27 weeks, compared to those with normal placental histology. However, in infants born after 27 weeks, placental inflammation showed a protective effect when compared to placental malperfusion. When interpreting the comparisons, it is important to consider the absence of a “real” control group, as emphasized by Suhas G. Kallapur and Alan H. Jobe, since preterm labor cannot be considered as a normal process in any case [45].

The absence of pathological findings in placental histology indicates that placental pathology was not the trigger for preterm labor. In this study, in 45% of cases, preterm labor occurred spontaneously with an unknown etiology, while 25% of cases were associated with potential inflammatory causes (e.g., ruptured membranes, clinical chorioamnionitis, and placental abruption) (see Supplementary Information 1). Even today, the mechanisms underlying preterm labor remain elusive in many cases. Furthermore, even in cases with placental pathology, delays in obtaining histology results limit their integration into real-time clinical management. This highlights the need for improvements in the process, allowing histological data to be utilized more effectively and contributing to a more personalized approach to care, with the potential to improve long-term outcomes.

## Limitations

The main limitation of this study is that, due to stratification by sex and gestational age groups, the sample size for some comparisons was reduced, particularly for infants born before 27 weeks of gestation. This could have significantly limited the statistical power and capacity to detect significant differences in this critical subgroup. Additionally, the observational nature of the study means that other unmeasured variables may have influenced the observed associations between placental histology and BPD development, making it difficult to establish direct causality.

Furthermore, placental histological findings were grouped into three categories to facilitate analysis and interpretation which may oversimplify the variety and complexity of placental abnormalities, with different etiopathogenic mechanisms, potentially leading to loss of nuanced information about how different placental pathologies might impact preterm birth and BPD outcomes.

The severity of placental lesions, which may also play an important role in neonatal outcomes, was not considered in this study and could further influence the observed associations.

In this study, birth weight *z*-scores were not included as a variable since they were considered a potential consequence of placental dysfunction, and their inclusion could introduce bias in the relationship between the exposure and the outcomes. However, their analysis could provide valuable insights into whether vascular abnormalities mediate their effects through impaired fetal growth. This represents a limitation of our current dataset but highlights an important avenue for further research.

Also, the inclusion of both singleton and multiple pregnancies in this study may have introduced heterogeneity into the sample, potentially leading to confounding factors such as varying risks of preterm birth and complications associated with multiple gestations. Future research may benefit from focusing exclusively on singleton pregnancies to achieve more homogeneous results and minimize such confounding variables.

## Conclusions

In our analysis of the preterm patient population, placental inflammation was found to increase the incidence of BPD, primarily by triggering extremely preterm births. However, it did not result in higher morbidity when comparing groups of similar gestational ages. In contrast, placental malperfusion exhibited sex-specific effects on lung development, with male infants born at or after 27 weeks GA showing greater susceptibility to BPD, independent of gestational age or mechanical ventilation. These findings underscore the importance of considering sex differences in the pathophysiology of BPD and the role of placental pathology.

## Supplementary Information

Below is the link to the electronic supplementary material.

Supplementary file1 (DOCX 35.8 KB)

## Data Availability

No datasets were generated or analysed during the current study.

## Abbreviations

*aOR*: Adjusted odds ratio
*AUC*: Area under the curve
BPD: Bronchopulmonary dysplasia grades 2–3
*CI*: Confidence interval
COVID 19: Coronavirus Disease 2019
GA: Gestational age
*IQR*: Interquartile range
MV: Mechanical ventilation
NICU: Neonatal Intensive Care Unit
NIH: National Institutes of Health
PMA: Postmenstrual age
SFBPD: Survival without BPD grades 2–3
