# Correction to: Impact of placental pathology on the risk of bronchopulmonary dysplasia in preterm infants: The role of gestational age and sex

**DOI:** 10.1007/s00431-025-06469-y

**Published:** 2025-09-27

**Authors:** C. Ramos-Navarro, R. Gregorio-Hernández, A. Pérez-Pérez, E. Rodríguez-Corrales, S. Vigil-Vázquez, M. Arriaga-Redondo, A. Merino-Hernández, M. Sánchez-Luna

**Affiliations:** 1 Neonatology Department, Gregorio Marañón University Hospital, Madrid, Spain; 2 Complutense University of Madrid, Madrid, Spain

**Correction to: European Journal of Pediatrics (2025) 184:211**

10.1007/s00431-025-06016-9

The chapter “Impact of placental pathology on the risk of bronchopulmonary dysplasia in preterm infants: The role of gestational age and sex,” written by Rodríguez‑Corrales E., Vigil‑Vázquez S., Arriaga‑Redondo M., Merino‑Hernández A. and Sánchez‑Luna M., was originally published without open access. Following the author’s/authors’ decision to opt for open access, the copyright of the chapter changed on September 3, 2025 to © The Author(s), 2025 and the chapter is now distributed under the terms of the Creative Commons license CC BY. Open Access funded by: CRUE-CSIC agreement with Springer Nature.

The original article has been corrected.

