# Supplementary material for: Impact of placental pathology on the risk of bronchopulmonary dysplasia in preterm infants: The role of gestational age and sex
Source: Eur J Pediatr. 2025 Feb 26;184(3):211. doi: 10.1007/s00431-025-06016-9 (PMC12476302; doi:10.1007/s00431-025-06016-9)
Supplement: Supplementary file 1 — Supplementary file1 (DOCX 35.8 KB) [file 431_2025_6016_MOESM1_ESM.docx]

**Supplementary Information 1. Potential Causes of Preterm Birth in Cases with No Pathological Findings in Placental Histology (n = 235) and in Cases Without Placental Histology (n = 229).** Data are expressed as the number of cases and proportion [n (%)].

| **Causes in no pathological findings (n 235)** | **N (%)** |
| --- | --- |
| **Spontaneous preterm birth** | **106 (45.10%)** |
| **Inflammatory Causes** | **59 (25.11%)** |
| - Clinical chorioamnionitis | 2 (0.85%) |
| - Prelabor rupture of membranes | 48 (20.43%) |
| - Placental abruption | 5 (2.13%) |
| - Maternal COVID-19 pneumonia | 4 (1.70%) |
| **Malperfusion** | **40 (17.02%)** |
| - Intrauterine growth restriction (IUGR) | 11 (4.68%) |
| - Hypertensive disorders of pregnancy | 9 (3.83%) |
| - Twin-to-twin transfusion syndrome | 16 (6.81%) |
| - Hemorrhage | 4 (1.70%) |
| **Multiple Birth** | **11 (4.68%)** |
| - Triplets | 6 (2.55%) |
| - Quintuplets | 5 (2.13%) |
| **Fetal Causes** | **14 (5.96%)** |
| - Loss of fetal well-being | 4 (1.70%) |
| - Hydrops | 1 (0.43%) |
| - IUGR sibling | 6 (2.55%) |
| - Umbilical cord prolapse | 3 (1.28%) |
| **Maternal Causes** | **5 (2.13%)** |

| **Causes in no performed histology (n229)** | **N (%)** |
| --- | --- |
| **Spontaneous preterm birth** | **85 (37.10%)** |
| **Inflammatory Causes** | **76 (33.20%)** |
| - Clinical chorioamnionitis | 10 (4.40%) |
| - Prelabor rupture of membranes | 60 (26.20%) |
| - Placental abruption | 3 (1.30%) |
| - Maternal COVID-19 pneumonia | 3 (1.30%) |
| **Malperfusion** | **46 (20.10%)** |
| - Intrauterine growth restriction (IUGR) | 11 (4.80%) |
| - Hypertensive disorders of pregnancy | 16 (7.00%) |
| - Twin-to-twin transfusion syndrome | 7 (3.10%) |
| - Hemorrhage | 12 (5.20%) |
| **Multiple Births (Triples)** | **3 (1.30%)** |
| **Fetal Causes** | **7 (3.10%)** |
| - Loss of fetal well-being | 3 (1.30%) |
| - IUGR sibling | 2 (0.90%) |
| - Umbilical cord prolapse | 2 (0.90%) |
| **Maternal Causes** | **2 (0.90%)** |
| **Deliveries at External Hospitals** | **10 (4.40%)** |

**Supplemental Information 2: Patient Flow Diagram (2012–2023). Cohort of Infants <32 Weeks of GA and Placental Histology Categories.** Data expressed as absolute frequencies and percentages n,(%). Abbreviations: GA, Gestational Age; w, weeks.

**Supplementary Information 3. Survival Without BPD (SFBPD) by Gestational Age: Weekly Comparisons and**

**Supplementary Information 4. Comparison of Area Under The Curve (AUC) for Survival without BPD 2-3 (SFBPD) and Mortality Across Gestational Ages in weeks (GA)**

| **GA** | **SFBPD** | **Mortality** |
| --- | --- | --- |
|  | AUC(95%IC) | AUC(95%IC) |
| 24wGA | 0.52 (0.45-0.69) | 0.53 (0.43-0.63) |
| 25wGA | 0.61 (0.54-0.68) | 0.69 (0.59-0.79) |
| 26wGA | 0.71 (0.64-0.78) | 0.78 (0.70-0.87) |
| 27wGA | **0.75 (0.71-0.79)** | **0.84 (0.77-0.90)** |
| 28wGA | 0.74 (0.70-0.77) | 0.80 (0.73-0.83) |
| 29wGA | 0.71 (0.69-0.75) | 0.73 (0.66-0.80) |
| 30wGA | 0.69 (0.65-7.24) | 0.69 (0.62-0.76) |
| 31wGA | 0.61 (0.57-0.64) | 0.62 (0.45-0.70) |
